# Understanding gerontological nursing competence among nursing students in higher vocational colleges: a cross-sectional study

**DOI:** 10.3389/fpubh.2025.1626565

**Published:** 2025-11-04

**Authors:** Qiu Chen, Ke Wang, Ying Gao, Jun Shen, QianYing Jia, XinXia Wang

**Affiliations:** ^1^Chongqing Emergency Medical Center, Chongqing University Central Hospital, Chongqing, China; ^2^The First Affiliated Hospital of Chongqing Medical University, Chongqing, China; ^3^University of Wollongong, Wollongong, NSW, Australia

**Keywords:** gerontological nursing, nursing student, nursing competence, cross-sectional study, nursing education

## Abstract

**Background:**

In light of the global aging trend, society requires an adequate supply of nursing personnel to deliver care services to older people. Concurrently, nursing staff involved in older people’s care must possess sufficient theoretical knowledge, practical skills, and professional competence, which are critical determinants of nursing quality.

**Aims and hypotheses:**

To investigate the level of gerontological nursing competence among nursing students in higher vocational colleges and identify factors associated with it. Specifically, we hypothesize that nursing students with more gerontological nursing education and positive interaction experiences with older people will demonstrate higher levels of competence in gerontological nursing.

**Methods:**

A descriptive cross-sectional study was conducted from April to July 2024, including nursing students from two higher vocational colleges in Chongqing, China.

**Results:**

1825 questionnaires were issued, and 1777 valid questionnaires were collected, with an effective recovery rate of 97.37%, and the total score of 1777 nursing students is (111.84 ± 23.01), which suggests that the overall gerontological nursing competence of nursing students is at the upper-middle level. Linear regression analysis revealed that grade, gender, being an only child at home, the relationship with grandparents, involvement in geriatric-related activities, prior experience in gerontological nursing education, and interest in gerontological nursing significantly positively influenced the competence of nursing students (*p* < 0.05).

**Conclusion:**

The majority of nursing students showed moderate to high levels of competence in gerontological nursing, which is essential for forming positive attitudes toward gerontological nursing. Statistical analysis revealed that gerontological nursing competence ratings were higher among nursing students who had received education in gerontological nursing, engaged in gerontological nursing practice, maintained positive social relationships with their grandparents, and exhibited an interest in gerontological nursing. This indicates that achieving a high degree of competency in gerontological nursing requires extensive theoretical knowledge, advanced understanding of gerontological nursing research, and a recognition of aging culture.

## 1 Introduction

Research indicates that the number of older people is increasing in countries worldwide. However, countries are at different stages of the longevity trend (1). According to International Population Reports, the global population aged 65 years and older already accounts for 8.5% (617 million) of the world’s population and is expected to increase to nearly 17% (1.6 billion) by 2050 (2, 3). China has long entered an aging society, and according to the seventh population census, the proportion of the older population has reached 18.7% as the aging process accelerates (4). As China faces a significant surge in its older population, with an anticipated 400 million people over 65 by 2050 and 150 million people aged 80 and above (5), the demand for gerontological nursing expertise becomes more urgent. To address this challenge, a comprehensive nursing education system is essential to equip students to address the various health needs of the aged population.

Studies have shown that as people age, the physiological functions of older people deteriorate, the likelihood of chronic diseases and accidental injuries escalates, and the prevalence of impairment may persistently rise (6). In China, 74.2% of those aged over 60 have been diagnosed with at least one common chronic illness. The population of older people with disabilities and those who are semi-disabled will continue to rise, accordingly increasing the demand for nursing and life support services (7), as well as for caregivers possessing expertise in gerontology. However, the existing educational and evaluative framework in nursing is inadequate, and the absence of professional training for caregivers hinders their ability to address the complex needs of nursing, which could compromise the well-being of older people.

With advancements in social development, the dimensions of gerontological nursing needs are expanding, becoming increasingly diversified and individualized (8, 9). This places higher demands on the professionalism and overall competence of nursing staff, further highlighting the importance of systematic training and evaluation. It can be said that the tremendous increase in the proportion of older people poses several challenges to gerontological nursing education. Therefore, while society requires an adequate number of nursing staff to deliver care services, it is equally important that these personnel possess comprehensive theoretical knowledge, advanced practical skills, and an excellent level of professionalism. In China, nursing students in higher vocational colleges are the main workforce for gerontological nursing, but there is little research regarding the competency levels of these students.

Clinical competence is defined as the knowledge, skills, attitudes, and abilities to practice safely and effectively without the supervision of others (10), while gerontological nursing competence covers a broad spectrum of nursing practice and includes more than just the skills and knowledge required to provide quality and abundant bedside care. It also focuses on the leadership, need for information, coordination of services and supports, and community engagement needed to improve nursing for the older people across diverse settings (11). Gerontological nursing is defined by the American Nurses Association (ANA) as an evidence-based nursing specialty that focuses on the aging process and the protection and promotion of health and function. The World Health Organization defines gerontological nursing as the provision of nursing care in various care settings for older people as a member of a multidisciplinary health and social care team. It can be concluded that nursing for older people should have sufficient gerontological nursing competence to meet the various requirements. Consequently, to guarantee that nurses exhibit gerontological nursing competence, it is essential to enhance nursing education and the evaluation of such ability (10, 12).

Nursing education in China is categorized as basic nursing education, postgraduate nursing education, and continuing nursing education. Basic nursing education mainly includes secondary nursing education (secondary school) and higher nursing education (including college and undergraduate students), where the curriculum of both secondary technical schools and colleges is a three-year program (13). The purpose of basic nursing education is to prepare students for clinical nursing, community nursing, or follow-up education after graduation (14). Therefore, given the critical role of nurses in gerontological nursing, this study aims to assess the gerontological nursing competence of nursing students in higher vocational institutions and to investigate the factors influencing this competence.

## 2 Methods

### 2.1 Design

Cross-sectional studies have advantages in terms of cost, efficiency, and intergroup comparison. Given increasing nursing demands in an aging population, cross-sectional research helps rapid data collection and is more appropriate for showing current situations and conducting multifactorial analysis to assess the simultaneous impact of various factors on gerontological nursing competence. A cross-sectional study was done from April to July 2024 among nursing students at two distinct higher vocational colleges in Chongqing, China. Nursing students in higher vocational institutions in China must complete a three-year program, which includes an internship lasting eight to twelve months in a teaching hospital in the final year. Finally, the first and second-year nursing students were included in this study.

### 2.2 Setting and sample

To better match the research objectives, select representative institutions or groups, reduce resource consumption, and control data collection costs, a purposive sampling method was used to select two higher vocational colleges in Chongqing as research institutions, and nursing students in their first and second years were chosen as study participants. The nursing curriculum at the two vocational colleges is practice-oriented, with a joint teaching hospital and a varied and high-quality student body, making them both representative and exemplary vocational nursing institutions in southwest China. The inclusion criteria were (1) studying in full-time higher vocational colleges and (2) voluntarily participating in the study and signing the informed consent. The exclusion criteria were (1) refusal to engage in the study, (2) inability to access the online survey due to technical issues, and (3) incomplete or inconsistent responses, or failure to understand the survey language.

### 2.3 Sample size calculation

According to the rough sample size estimation method proposed by Kendall, compared with the largest number of variables, the sample size should be 5 to 10 times, and then expanded by 10%. There are 12 variables in the general data questionnaire of this study, 35 variables in the gerontological nursing competence evaluation scale, and all the study variables are 47. According to the sample size calculation formula: N = (variable × multiple) × (1 + 10%), the sample size of this study is at least 259 cases.

### 2.4 Instrument

The study makes use of a self-administered questionnaire that has three sections. The first section is the questionnaire introduction, which describes the goal of the study, the requirements for completing the questionnaire, and assures participants that their personal information will be kept private and used only for this study. The second section includes age, gender, grade level, place of residence, whether they were the only child at home, and additional demographic data. Our research group developed an evaluation scale of gerontological nursing competence for students at higher vocational institutions, which makes up the third section.

In 2020, our research team developed an evaluation scale for gerontological nursing competence among nursing students in higher vocational institutions by literature analysis, expert group discussions, and a pre-survey. The scale contains four dimensions, including gerontological nursing practice ability (11 items), professional development and guidance ability (8 items), communication and cooperation ability (8 items), and legal and ethical quality (8 items), a total of 35 items. A 5-point Likert scoring method was used for each item, ranging from “not competent” to “very competent,” with a total score ranging from 35 to 175. A higher score indicates superior self-assessment competence among students. The Cronbach’s coefficient alpha for the scale was 0.971, with individual dimensions measuring 0.940, 0.931, 0.934, and 0.918, while the retest reliability coefficient was 0.904. The χ^2^ degree of freedom ratio was 4.911, and the mean square and square root of the progressive residual were 0.071. The values of the I-CVI ranged from 0.800 to 1.000, and the S-CVI was 0.930.

### 2.5 Data collection

The questionnaires were distributed and completed online. Before the survey, the director of the study will appoint a person in charge of each of the two survey institutions, and they will explain the purpose, main content, significance, and requirements for completing in the questionnaire to the nursing students, and then distribute the questionnaire links. After obtaining the teacher’s authorization, the person in charge will inform participants about the research inclusion criteria and their right to refuse participation, followed by the distribution of the questionnaire links. The questionnaire will take around 15 to 20 min for nursing students who meet the eligibility and consent requirements to complete and submit and the investigator will consistently monitor the completion of the questionnaire and ensure quality control by discarding incorrect submissions. Finally, a total of 1825 questionnaires were issued and 1777 valid questionnaires were collected, with an effective recovery rate of 97.37%.

### 2.6 Ethical consideration

This study was approved by the Ethics Committee of Chongqing Emergency Medical Center. Participants indicate their willingness to participate in this study and complete the informed consent form. The investigator also states that the participants’ personal information will be kept confidential and used only for this study.

### 2.7 Data analysis

IBM SPSS 23.0 was used to analyze the data. After checking the normality of the data, quantitative data conforming to normal distribution was expressed as mean ± standard deviation (x ± s), while quantitative data not conforming to normal distribution was expressed as median (quartile) [M (P25, P75)]. Independent sample t-test, one-way ANOVA, and Spearman correlation analysis were used to analyze the differences in nursing competence of students with varying characteristics. In addition, with the score of the gerontological nursing competence evaluation scale as the dependent variable and the meaningful variables in the single factor analysis as the independent variable, multiple linear regression was used to analyze the influencing factors of the gerontological nursing competence of students. *p* < 0.05 was considered statistically significant.

## 3 Results

### 3.1 Demographic characteristics

Demographic characteristics see Table 1.

**Table 1:** Demographic characteristics of the nursing students (*N* = 1,777).

Sociodemographic characteristics	Mean (SD)/n (%)
Age	18.89 (1.005)
Gender	
Male	184 (10.4)
Female	1,593 (89.6)
Grade	
Grade one	974 (54.8)
Grade two	803 (45.2)
Place of residence	
Rural	1,275 (71.8)
Urban	502 (28.2)
The only-child at home	
Yes	460 (25.9)
No	1,317 (74.1)
Experience of being taken care of	
Parents or single parent	1,234 (69.4)
Grandparents	450 (25.3)
Other	93 (5.2)
Lived with older family members	
Yes	1,558 (87.7)
No	219 (12.3)
Duration of living with older people	
≦1 year	214 (12.0)
2 ~ 3 year	368 (20.7)
≧4 year	1,195 (67.2)
Participated in older people -related activities	
Never	962 (54.1)
Occasionally	755 (42.5)
Often	60 (3.4)
Prior experience of gerontological nursing education	
Yes	749 (42.1)
No	1,028 (57.9)
Interest in gerontological nursing	
Not interested at all	20 (1.1)
Not interested in	102 (5.7)
Normal	947 (53.3)
Interested in	602 (33.9)
Be very interested in	106 (6.0)
Relationship with grandparents	
Very bad	2 (0.1)
Bad	6 (0.3)
Normal	257 (14.5)
Good	699 (39.3)
Very good	813 (45.8)

### 3.2 The scores of gerontological nursing competence of students

The scores of gerontological nursing competence of students see Table 2.

**Table 2:** The scores of gerontological nursing competence of students (*N* = 1,777).

Dimensions	Score (x ± s)
Gerontological nursing practice ability	30.85 ± 8.45
Professional development and guidance ability	24.60 ± 6.25
Communication and collaboration ability	27.36 ± 5.80
Legal and ethical quality	29.02 ± 6.07
Total	111.84 ± 23.01

### 3.3 Comparison of gerontological nursing competence by student characteristics

Comparison of gerontological nursing competence by student characteristics see Table 3.

**Table 3:** Comparison of gerontological nursing competence by student characteristics (*N* = 1,777).

Variables	Score (Mean, SD)	t/F	*p*	Cohen’s *d*	Partial *η*^2^
Grade				0.338	
Grade one	108.38, 23.03	−7.084	<0.01		
Grade two	116.04, 22.29				
Gender				0.214	
Male	116.24, 25.99	−2.46	0.015		
Female	111.33, 22.59				
Place of residence				0.01	
Rural	111.78, 22.78	−0.183	0.855		
Urban	111.99, 23.60				
The only-child at home				0.158	
Yes	114.53, 24.81	−2.769	0.006		
No	110.90, 22.28				
Experience of being taken care of					0.003
Parents or single parent	112.17, 22.87	2.916	0.054		
Grandparents	112.10, 23.01				
Other	106.24, 24.33				
Lived with older family members				0.184	
Yes	112.36, 23.04	−2.547	0.011		
No	108.14, 22.51				
Duration of living with the older people					0.007
≦1 year	108.14, 22.41	6.252	0.002		
2 ~ 3 year	109.75, 23.70				
≧4 year	113.15, 22.80				
Participated in older people-related activities					0.065
Never	107.81, 22.44	61.713	<0.01		
Occasionally	114.96, 21.85				
Often	137.08, 24.46				
Prior experience of gerontological nursing education				0.21	
Yes	117.38, 22.91	−8.859	<0.01		
No	107.80, 22.24				
Interest in gerontological nursing					0.092
Not interested at all	96.80, 26.95	45.01	<0.01		
Not interested in	99.77, 20.57				
Normal	108.57, 21.31				
Interested in	115.78, 22.46				
Be very interested in	133.10, 24.76				
Relationship with grandparents					0.052
Very bad	99.00, 90.50	24.249	<0.01		
Bad	104.00, 23.41				
Normal	101.98, 21.32				
Good	109.78, 20.44				
Very good	116.82, 24.13				

### 3.4 Multiple regression analysis of gerontological nursing competence in students

Multiple regression analysis of gerontological nursing competence in students see Table 4.

**Table 4:** Multiple regression analysis of gerontological nursing competence in students (*N* = 1,777).

	Unstandardized coefficients		Standardized coefficients	t	*p*	Collinearity statistics	
	*B*	Standard error	*Beta*			VIF	Tolerance
Variables	55.378	10.409	-	5.32	<0.01	-	-
Age	−0.082	0.553	−0.004	−0.148	0.883	1.24	0.806
Grade	3.95	1.189	0.085	3.321	<0.01	1.407	0.711
Gender	4.812	1.659	0.064	2.9	<0.01	1.026	0.975
The only-child at home	2.777	1.15	0.053	2.416	0.016	1.018	0.982
Lived with older family members	−0.175	2.234	−0.002	−0.078	0.938	2.165	0.462
Duration of living with older people	0.275	1.072	0.008	0.257	0.797	2.252	0.444
Relationship with grandparents	4.086	0.744	0.13	5.49	<0.01	1.188	0.842
Participated in older people-related activities	5.217	0.936	0.128	5.576	<0.01	1.116	0.896
Prior experience of gerontological nursing education	5.683	1.112	0.122	5.109	<0.01	1.211	0.825
Interest in gerontological nursing	6.731	0.725	0.214	9.287	<0.01	1.132	0.883
*R^2^*	0.169						
Adjusted R^2^	0.164						
*F*	*F* (10,1766) = 35.892,*p* = 0.000						
D-W	1.943						

## 4 Discussion

The results of this study show that the total score of gerontological nursing competence of 1777 vocational nursing students is (111.84 ± 23.01), which suggests that the overall gerontological nursing competence of students is at the upper-middle level, which is similar to the conclusion of Tohmola et al. (12). In this study, the dimension of gerontological nursing practice is (30.85 ± 8.45), professional development guidance is (24.60 ± 6.25), communication and cooperation is (27.36 ± 5.80), and legal ethics is (29.02 ± 6.07), which suggests that nursing students are more confident in gerontological nursing practice, communication and cooperation ability, legal and ethical quality, but weaker in professional development guidance ability. The dimension of professional development guidance in this study mainly includes the provision of professional nursing care, health counseling, individual life planning for older people or their families, as well as self-exploration of knowledge related to care, and a focus on nursing development and related topics. Analyzing the results of this study, the low scores in professional development guidance ability may be attributed to nursing students’ completion of gerontological nursing knowledge and assessment, yet their inability to effectively integrate theory and practice due to insufficient practical opportunities. Furthermore, the scores in communication and cooperative skills, as well as legal and ethical standards, are relatively elevated, potentially due to the absence of interactions with real patients and their families throughout class, leading to an optimistic attitude toward communication. A study by Ferretti-Rebustini et al. (15) once pointed out that gerontological nursing competence covers multiple dimensions, but in terms of levels, the most important competence may be practice competence, because the foundation of gerontological nursing depends on clinical competency, which can determine the need for action and follow-up through comprehensive assessment of patients’ problems, while combining clinical judgment and critical thinking to immediately recognize and appropriately manage common conditions in older people. Consequently, in gerontological nursing education, educators and clinical instructors should collaborate on developing strategies for integrating gerontological nursing content, addressing deficiencies in current curricula and practices to enhance nursing students’ competencies. During the instructional process, students may improve their competence in addressing real-world nursing problems through the combination of situational simulation training and case-based teaching, particularly in managing prevalent health issues among older people.

In our study, it was observed that gerontological nursing competence was significantly correlated with years of college and gender (*p* < 0.01, *p* = 0.015). It was also observed that second-year nursing students outperformed first-year students (*p* < 0.01). This is different from the findings of Alqahtani et al., who reported in their study that first-year students had better competence in caring for the older people (16), but similar to the findings of Cheng et al. (17). In the two study institutions, second-year nursing students have completed a specialized gerontological nursing module, which includes nearly 40 h of theoretical instruction and a two-week clinical internship, in contrast to first-year students. This type of curricular variation may contribute to the observation of higher scores among second-year students. The following studies should investigate how the various elements of gerontological nursing curricula influence students’ attitudes and competencies, including course length and clinical practice arrangements.

In addition, male nursing students achieved a higher score (116.24 ± 25.99). It suggests that male nursing students possess a superior self-assessment of gerontological nursing competence, exhibit more favorable attitudes toward the gerontological nursing profession, and may be more likely to pursue gerontological nursing-related employment post-graduation. An integrative review (18) indicated that the different results regarding male and female attitudes toward gerontological nursing across various geographical regions can be attributed to cultural differences and social context, as well as methodological factors such as sample size or bias caused by an inadequate representation of male nurses. In this study, considering that with the increasing social emphasis on gender equality and cognitive development, men are gradually stepping into traditionally female-dominated professions like nursing, male students may receive more attention or support in education, and are often encouraged to show confidence and leadership, which may contribute to their higher self-competence evaluation. Moreover, men are a minority in the nursing profession, and this self-perception may compel them to exert greater effort to demonstrate their expertise and competence in fields traditionally dominated by women. Additionally, it is worth noting that other factors such as career choice motivations and individual personality traits may also influence these gender differences in self-assessed competence. For instance, male nursing students may choose this profession with clearer career advancement goals or stronger personal interest in specialized clinical areas like gerontological care (19), which could enhance their engagement and confidence. According to social role theory, gender stereotypes derive from the discrepant distribution of men and women into social roles both in the home and at work. In the workplace, women have tended to be employed in people-oriented, service occupations rather than things-oriented, competitive occupations, which have traditionally been occupied by men (20). From this, we can infer that personality traits such as higher levels of self-confidence and leadership and assertiveness typically reported among male students could contribute to higher self-evaluation scores.

Our research additionally revealed that prior relationships with grandparents positively influenced gerontological nursing competency, consistent with other findings (21). Approximately 80% of nursing students claimed a better connection with their grandparents, whereas merely 8% reported a deteriorated relationship. From a cultural and social perspective, it is evident that China has historically promoted the concept of filial piety, which has influenced nursing students from a young age, fostering positive attitudes toward older people and resulting in minimal prejudices and stereotypes (14). The difference is that cultural concepts such as long-lived people and those with dementia being “wizards” or “wizards” have been deeply rooted in Ghanaian society for many years. These negative cultural concepts have deeply cultivated wrong notions about aging among nursing students, which may affect their ability to care for the older people (22). Kydd et al. (23) emphasize that the integration of gerontology and cultural competence into the nursing curriculum is essential for the future development of nursing education. Nursing students should be provided with opportunities to reflect on their personal and cultural values. As nursing educators, it is important to facilitate a process through which students critically examine their personal and professional value systems, explore how these values influence their understanding of diverse cultural beliefs, and develop intergenerational interaction and cross-cultural empathy by engaging in multicultural older people care experiences. In the current study, we primarily focused on the Chinese cultural context, particularly the influence of filial piety. The complex role of cultural background in the cultivation of gerontological nursing competence should be further explored in future studies. Evidence from this study suggests that negative experiences in past social relationships with grandparents may be associated with a self-protective mindset among nursing students, which correlates with less positive attitudes toward gerontological nursing and reduced learning initiative. Given these associations, it is advisable for nursing educators to pay attention to students reporting such experiences and consider incorporating targeted educational strategies aimed at fostering empathy and positive perceptions toward older people. Developing individualized gerontological nursing education and training programs might help support these students in forming a more comprehensive and accurate understanding of gerontological nursing (24).

In this study, 42.1% of nursing students reported that they had engaged in gerontological nursing education, and 45.9% of nursing students reported that they had participated in social practice activities related to older people. Statistical analyses indicated that there was a significant difference between this group of students and those who had not participated in this type of study or activity, which is consistent with the results of previous studies (17). A certain amount of gerontological nursing education, experiential learning, and related volunteer experiences would enhance students’ compassion, empathy, and respect for the aged population. As the Knowledge, Beliefs, and Behavior (KAB) (25) model suggests, health knowledge and information are integral to forming positive and accurate beliefs and attitudes and changing health-related behaviors. Beliefs and attitudes are the driving force behind behavior change (26). Consequently, as nursing educators, it is essential to reform teaching methodologies and content tailored to the target audience, enhance the contextualization of the curriculum, and incorporate additional learning experiences regarding older people to improve nursing students’ beliefs and competencies in gerontological nursing.

The findings of this study showed that students with different levels of interest in gerontological nursing had statistically significant differences in their scores on the competency evaluation. The difference is seen in that students exhibiting a greater interest in gerontological nursing attained superior competency evaluation scores. It can also be interpreted that there was a correlation between higher interest and higher willingness to participate. Desire to practice is considered to be an ideological disposition and behavioral motivation to practice, which may influence their actual career choices (27). Therefore, the degree of nursing students’ interest in gerontological nursing will influence their employment orientation post-graduation, making it crucial to enhance this interest through the teaching process (28). Furthermore, it is essential to consider nursing students’ motivation to engage in practice and to offer systematic, standardized, and comprehensive guidance for their career planning, thereby enhancing their employability and professionalism. We believe that engaging with experienced gerontological nursing professionals and establishing early interactions with older people may more effectively foster students’ interest, such as by inviting experts to conduct seminars, career-sharing sessions, and community volunteer initiatives.

### 4.1 Limitation

This research possesses certain limitations. The data for this cross-sectional study was exclusively derived from nursing students at two vocational colleges in Chongqing, resulting in a limited sample size and composition, as well as a structural imbalance between male and female students in the study population. Consequently, the present findings may not be generalizable to all nursing students in China, requiring a larger sample size. Secondly, due to the voluntary nature of survey participation, selection bias may further restrict generalizability, and participants’ self-reported replies may not accurately reflect their actual behavior. The notion of filial piety in Chinese culture and its significance in gerontological nursing may restrict the generalizability of the findings to diverse cultural contexts or demographics. When conditions permit additional investigation, this study may be expanded to a cross-cultural scope to assess the gerontological nursing competence of nursing students from various cultural backgrounds and the factors influencing it. Furthermore, the inclusion of objective evaluation metrics would improve the comprehensiveness of the assessment, so expanding both the scope and depth of the research.

## 5 Conclusion

This study revealed that most nursing students had moderate to high levels of competency in gerontological nursing, which is crucial for cultivating favorable attitudes about the profession. Statistical analysis revealed that higher ratings of gerontological nursing competence among students correlated with their education in gerontological nursing, participation in gerontological nursing practice, positive social relationships with grandparents, and interest in gerontological nursing. This indicates that achieving a high level of competence in gerontological nursing requires extensive theoretical knowledge in the discipline, advanced understanding of research developments relevant to gerontological nursing, and an appreciation of the culture of aging. It can be concluded that practical teaching in gerontological nursing should be incorporated early in the curriculum of gerontological nursing programs to guarantee that students attain enough clinical competence. Educators must examine the diverse educational backgrounds and life experiences of students, acknowledge their emotional and educational requirements, and facilitate their learning of gerontological nursing in an appropriate way. In addition, longitudinal research will help to understand the dynamic development process of nursing competence, cross-cultural comparative research will expand the applicability of the conclusions, and educational intervention research can promote the progress of global nursing education while verifying different educational strategies. In the future, these studies will help to lay a more solid foundation for cultivating qualified professionals in gerontological nursing.

## Data Availability

The original contributions presented in the study are included in the article/supplementary material, further inquiries can be directed to the corresponding author/s.
